# Sensorimotor Learning Biases Choice Behavior: A Learning Neural Field Model for Decision Making

**DOI:** 10.1371/journal.pcbi.1002774

**Published:** 2012-11-15

**Authors:** Christian Klaes, Sebastian Schneegans, Gregor Schöner, Alexander Gail

**Affiliations:** 1 Bernstein Center for Computational Neuroscience, German Primate Center – Leibniz Institute for Primate Research, Göttingen, Germany; 2 Division of Biology, California Institute of Technology, Pasadena, California, United States of America; 3 Institute for Neural Computation, Ruhr University Bochum, Bochum, Germany; Indiana University, United States of America

## Abstract

According to a prominent view of sensorimotor processing in primates, selection and specification of possible actions are not sequential operations. Rather, a decision for an action emerges from competition between different movement plans, which are specified and selected in parallel. For action choices which are based on ambiguous sensory input, the frontoparietal sensorimotor areas are considered part of the common underlying neural substrate for selection and specification of action. These areas have been shown capable of encoding alternative spatial motor goals in parallel during movement planning, and show signatures of competitive value-based selection among these goals. Since the same network is also involved in learning sensorimotor associations, competitive action selection (decision making) should not only be driven by the sensory evidence and expected reward in favor of either action, but also by the subject's learning history of different sensorimotor associations. Previous computational models of competitive neural decision making used predefined associations between sensory input and corresponding motor output. Such hard-wiring does not allow modeling of how decisions are influenced by sensorimotor learning or by changing reward contingencies. We present a dynamic neural field model which learns arbitrary sensorimotor associations with a reward-driven Hebbian learning algorithm. We show that the model accurately simulates the dynamics of action selection with different reward contingencies, as observed in monkey cortical recordings, and that it correctly predicted the pattern of choice errors in a control experiment. With our adaptive model we demonstrate how network plasticity, which is required for association learning and adaptation to new reward contingencies, can influence choice behavior. The field model provides an integrated and dynamic account for the operations of sensorimotor integration, working memory and action selection required for decision making in ambiguous choice situations.

## Author Summary

Decision making requires the selection between alternative actions. It has been suggested that action selection is not separate from motor preparation of the according actions, but rather that the selection emerges from the competition between different movement plans. We expand on this idea, and ask how action selection mechanisms interact with the learning of new action choices. We present a neurodynamic model that provides an integrated account of action selection and the learning of sensorimotor associations. The model explains recent electrophysiological findings from monkeys' sensorimotor cortex, and correctly predicted a newly described characteristic pattern of their choice errors. Based on the model, we present a theory of how geometrical sensorimotor mapping rules can be learned by association without the need for an explicit representation of the transformation rule, and how the learning history of these associations can have a direct influence on later decision making.

## Introduction

Actions beyond simple reflexes are generally not the direct consequence of a sensory input. Instead, the association of a specific sensory input with an appropriate action has to be learned from experience, and depends on the behavioral context. Often these context-dependent associations can be described in terms of a general mapping rule. In most situations, subjects can choose among more than one associated action. This requires a process for action selection, a form of decision making. We propose that a reward-based learning mechanism for forming new sensorimotor associations is integrated in the action selection system. Through this integration in a common neural substrate, the learning history directly influences the decision process.

While traditional psychological theories tended to view decision making as the outcome of a higher cognitive process which is separate from perception and action [1], more recent neurophysiologically motivated ideas emphasize the integrative nature of sensorimotor processing and action selection [2]–[5]. Several cortical areas form frontoparietal networks for making goal-directed saccades, like the lateral intraparietal area (LIP) and the frontal eye fields (FEF), or goal-directed reaches, like the parietal reach region (PRR) and the dorsal premotor cortex (PMd) [6]–[9]. At the same time, neurons in these areas show signatures of valuation and selection of action, since their neural responses are modulated by the subject's choice preference based on reward expectancy or other decision variables [5], [10]–[19].

We present a dynamic neural field (DNF) model that unifies the processes of sensory integration, working memory formation, associative learning and action selection in a context-dependent mapping task. The model implements a reward-driven Hebbian mechanism that allows it to learn simple associative sensorimotor mappings from reward history. The model selects from a continuum of ‘behavioral’ options through an integrated competition process between potential action plans. This framework reflects the conceptual idea of integrated sensorimotor and decision processing [2], [20].

With this model, first, we explain adaptive decision behavior and its neural underpinnings in tasks which require rule-based selection of spatial motor goals. As an example, we use the model to mimic the behavioral and neural findings of a previous monkey experiment. In this experiment, the authors investigated the preparatory neural activity in situations in which in response to an ambiguous visual cue, two potential motor goals could be ‘freely’ chosen according to two different spatial transformation rules [5]. Varying reward contingencies lead to different choice behavior and neural activity patterns in this experiment. Previous models of decision making did not utilize learning in decision tasks with ambiguous choice situations, hence could not adapt to different reward contingencies. Conceptually, they either did not implement neural-inspired mechanisms of sensorimotor mapping, like threshold-models of decision making (see [21] for review), or were limited to solve predefined target-selection tasks [2]. Other models, which implemented sensorimotor association learning, did not investigate decision making in ambiguous situations [22]–[24]. Second, we used our model to make predictions about specific patterns of choice errors in a generalization task. We tested the predictions which result from these assumptions in an additional behavioral monkey experiment.

Our results provide support for two assumptions, which are more general than the specific examples for which we directly demonstrate the suitability of our approach. The first assumption regards the neural mechanism underlying context-specific “rule-based” spatial remapping in visuomotor tasks. It is in general unclear if rules that can be derived by abstraction from concrete examples are encoded as such in the monkey brain, or if instead the brain stores the individual underlying associations that constitute the rule. We propose that spatial mapping rules are learned, at least in our monkey experiments, by local associations. The nature of local associations limits the ability to generalize a mapping rule and imposes interactions between novel cues and already trained cue locations, which lead to specific patterns of choice errors. The second assumption regards the interaction of sensorimotor learning with adaptive choice behavior in action selection tasks. We propose that the same reward-driven Hebbian learning mechanism which allows learning of arbitrary stimulus-response mappings also contributes to adapting the choice behavior to changing probabilistic reward contingencies in a free-choice task, in addition to other biasing factors for adapting choice behavior. As an inevitable consequence, the learning and reward history influences the decision process, and biases the behavior in free-choice situations.

## Methods

### Ethics statement

This study was granted permission to carry out experiments on vertebrates by the Niedersächsische Landesamt für Verbraucherschutz und Lebensmittelsicherheit, No 33-9-42502-047-064/07. All animal work was conducted according to the German Animal Welfare Act and all experiments were conducted in conformity with the European Communities Council Directive of November 1986 (86/609/ECC).

### Rule-based motor-goal selection tasks

**Figure 1:**
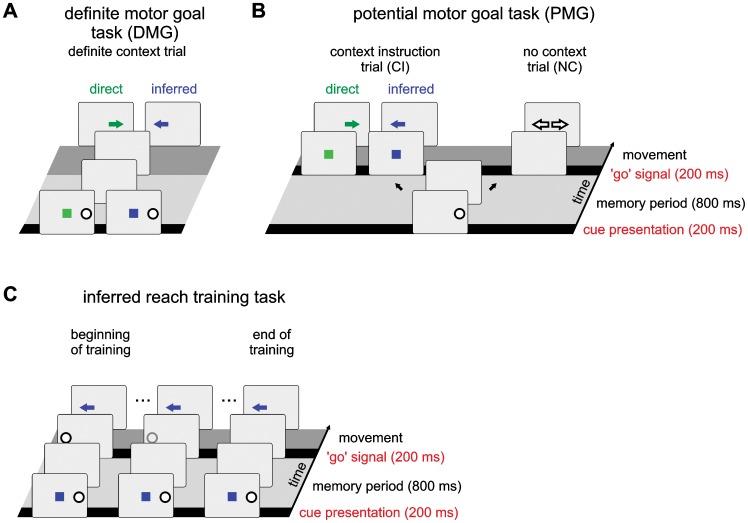
Structure of the context-dependent reach task that model and monkeys had to perform. In the beginning either a single spatial cue (PMG task, B) or a spatial and a contextual cue (DMG task, A) were presented, indicated by a white circle (spatial cue) and a colored rectangle (contextual cue). During the memory period no cue was shown. The ‘go’-signal indicated the subject to make a reach movement towards the goal, which was either at the same location as the spatial cue (direct trial; green) or at the diametrically opposite location (inferred trial; blue). In one part of the PMG trials the contextual cue was presented at the end of the memory period (PMG-CI), and in another part no contextual cue was shown at all (PMG-NC) and a free choice had to be made (see Methods). In the inferred reach training task (C), a second spatial cue (target cue) is shown at the end of the memory period to indicate the rewarded goal position. This cue is gradually faded out over many trials during the training.

Our approach addresses sensorimotor association learning and decision making in situations in which context-dependent remapping of a spatial sensory (e.g. visual) location onto different motor (e.g. reach) goals is required, and the mapping is achieved according to geometric transformation rules. Different variants of the task were employed in previous studies [5], [25]–[27] and are discussed in more detail below, but they all share the same basic structure (Figure 1): Two cues are presented, a spatial and a contextual cue, that together determine the rewarded goal location for a reach movement. The spatial cue is located at one of four equally spaced positions representing directions in the center-out workspace. The contextual cue can have two different colors and determines the mapping rule for the current trial. The mapping rule is either ‘direct’ (green), meaning that the rewarded motor goal is located at the same position as the spatial cue, or ‘inferred’ (blue), which means the rewarded goal is located in the direction opposite to the spatial cue. The reach movement has to be executed after a memory period upon a ‘go’-signal.

### Conceptual design of the neurodynamic model

**Figure 2:**
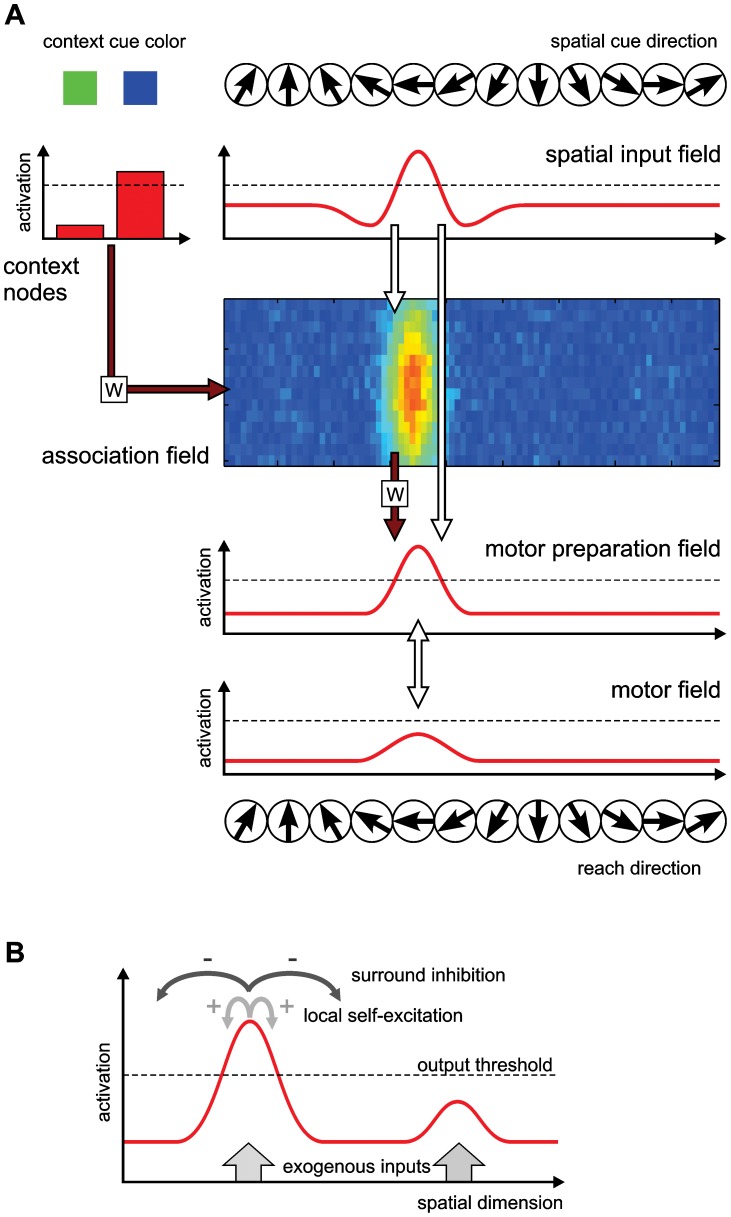
Model architecture and interactions in neural fields. (A) The model consists of four interconnected DNFs and a set of dynamic nodes. The spatial input field, motor preparation field, and motor field are one-dimensional fields that span the space of possible spatial cue/reach directions. The two-dimensional association field is defined over this directional space as well as a second dimension along which selectivity for the contextual cue develops. Its activation is shown color coded (red highest, blue lowest activation). The activation of the two context nodes is shown as a bar plot. Fixed projections between the fields are shown as white arrows; variable projections (that are subject to learning) are shown through dark red arrows with a weight matrix W. (B) Lateral interactions in DNFs, shown exemplarily for the motor preparation field. Exogenous input from other fields (indicated by grey arrows at the bottom) locally increases activation (red). Regions of high activation produce an output signal (the soft threshold of the sigmoid output function is indicated by the dashed line), which acts on other parts of the field and is also projected to other fields of the architecture. The lateral interactions consist of local excitatory connections and surrounding inhibitory connections, which together implement a soft competition between distant field regions. This creates a selection property in the field, promoting the formation of a single peak even for multi-modal input.

We use a model architecture that consists of multiple dynamic neural fields (DNF) to capture the neural processes underlying cue perception, working memory for visual locations, movement plan formation, and movement initiation (Figure 2A). Each DNF describes neural activation patterns at the population level. Its functional properties are determined by lateral interactions within each field (Figure 2B), which are predefined, and its connections to other fields in the architecture, which are partly plastic. This model is not intended to serve as a comprehensive and strict anatomical model, which is why we will have to refrain from drawing simple one-to-one links between individual DNFs and corresponding cortical areas. Nonetheless, the model architecture captures the general structure of spatial processing pathways in the primate frontoparietal cortex (see Discussion for a comparison to neurophysiology), and is largely analogous to a previous neurodynamic model that explicitly aimed to reproduce activation patterns in specific cortical areas [2]. The goal of the study is to emphasize general principles, which likely can be found in several sensorimotor subsystems, and to highlight these principles for a specific finding in specific cortical areas for which we have detailed knowledge.

The model is largely pre-structured in its inter-field connectivity (white projection arrows in Figure 2A). The pre-structuring allows it to perform basic functions without any initial training:

1. Selecting the spatial stimulus location as ‘default’ motor plan; this is realized by a direct topological connectivity between a spatial sensory input field and the motor-related fields (Figure 2A). This corresponds to the widespread tendency of subjects to direct their actions towards a salient stimulus, sometimes even involuntarily as in the case of saccades [28].
2. Selecting one reach plan out of several alternatives; this is realized by lateral inhibition and a winner-take-all dynamic (Figure 2B). This serves competitive action selection, i.e., the ability to ‘make a decision’.
3. Memorizing the last stimulus location in absence of the stimulus; this is realized by local excitation which can form self-sustained peaks of activation in DNFs (Figure 2B). This serves the function of a ‘working memory’.

These basic functions allow the model to produce a memory-guided reach directly towards a previously cued goal position as a default behavior. In addition to this, the model must be flexible enough to learn different spatial mappings from the spatial sensory input onto the motor output. This is achieved through additional plastic connectivity (red connections in Figure 2A) between input and output via a cue integration and association field. Plastic connections are adapted by a reward-driven Hebbian learning mechanism (see below).

### Dynamic neural fields

DNFs describe neural activation patterns through the evolution of continuous activation distributions over time, emphasizing the role of attractor states and instabilities [20], [29]. DNFs are based on the concept of population coding, in which a value along a certain feature dimension, e.g. the location of a visual stimulus or the endpoint of a planned movement, is represented through the distribution of activity within a population of neurons. These neurons have different tuning functions that sample the underlying feature space [30], [31]. Abstracting from the discrete spiking neurons, DNFs directly describe the activation distributions over the underlying feature space [32]–[35]. This activation distribution evolves continuously in time under the influence of external input and lateral interactions, governed by a differential equation of the form
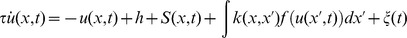



Here, 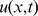 is the activation at time *t* for a position *x* along the underlying feature dimension, 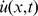 is its rate of change over time, which is scaled with a time constant 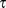, and 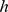 is the (negative) global resting level for the field activation. Any point in the field receives external input 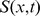, as well as endogenous input from other parts of the field. Furthermore, each point in the field is affected by additive noise 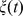, drawn from a normal distribution, that represents unspecific input and spontaneous activity. The lateral interactions are characterized by an interaction kernel 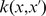, which consists of a local excitatory and a long-range inhibitory component. The lateral connectivity pattern reflects the mutual excitation between neurons with similar tuning curves and inhibition between those with dissimilar tuning curves. This interaction kernel is convolved with the output of the field, which is computed from the field activation via a sigmoid function,
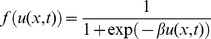



The field output is close to zero for low activation levels, rises around a soft threshold (arbitrarily placed at zero), and saturates for higher activations. The specific pattern of lateral interactions promotes the formation of localized peaks of activation as attractor states of the field dynamics (Figure 2B).

Depending on the interaction parameters (see Text S1, Table S1 and S2), different dynamic regimes can be achieved (for a quantitative analysis see [35]): With moderately strong local interactions, multiple simultaneous peaks can provide a representation of (multiple) current inputs that is stabilized against fluctuations. For stronger self-excitation (balanced by sufficient inhibition), peaks may become self-sustained in the absence of input, yielding a model of working memory (similar to the implementation with spiking neurons described by [36]). If strong global inhibition is present in a field, a competitive regime is created in which only a single peak can form, implementing a winner-take-all selection that is stabilized over time.

For numerical simulations, the conceptually continuous field dynamics have to be discretized in space and time. To perform comparisons with electrophysiological data, the field output at one point in the field is equated to the firing rate of neurons with corresponding selectivity profile. Evidence for a cortical organization that supports the neural field dynamic have been shown by [37].

### Model architecture

The dynamic model for context-dependent reaching consists of a set of interconnected DNFs and discrete nodes that can be organized into four levels: Perception (spatial and context input fields), memory and association (association field), movement planning (motor preparation field) and movement initiation (motor field), which are shown in Figure 2A. A complete formal description of the model with all parameter values is given in Text S1, Table S1 and Table S2.

A direct pathway from the *spatial input field* to the *motor preparation field* and further to the *motor field* implements a default sensorimotor mapping that is functional prior to any task-specific learning. The direct pathway comprises three DNFs defined over a one-dimensional space. In our case this space represents the angular direction (with circular boundary conditions) of either the location of the spatial cue (as direction from the central fixation point) or the direction of a reach movement in a center-out reach task. The projections between the fields along this direct pathway are topologically organized, that is, the output at a certain point in one field drives activation at the corresponding point (coding for the same direction) in another field, and to a lesser degree in the direct neighborhood of that point. The spatial input field features relatively weak local interactions to form a stabilized representation of a currently presented spatial cue. It projects in a topological fashion onto the motor preparation field. The motor preparation field has moderate local excitatory and global inhibitory interactions, producing a soft competition behavior between different regions of the field. While these competitive interactions promote the concentration of activation in a single region, they still allow multiple activation peaks to exist simultaneously if they are driven by multiple localized inputs.

The motor preparation field in turn reciprocally projects to the *motor field* in a topological manner. The motor field itself features stronger self-excitation and global inhibition, producing a strong selection behavior that only allows a single stabilized activation peak to prevail. The motor field is held at a low resting level during most of the time, so that it cannot form a peak from the motor preparation field's input alone. Only after the ‘go’-signal has been provided, the motor field is globally excited and an activation peak can form, simulating a gating mechanism for movement initialization. Similar gating mechanisms have been described for saccade generation [38]. When a peak has formed in the motor field, it projects back to the motor preparation field, such that the actually selected motor plan is reinforced in that field and others are suppressed.

An additional indirect pathway from the spatial input to the motor preparation field runs through the *association field*. This field spans two dimensions. The first dimension of the association field corresponds to the angular spatial representation also used in the spatial input field, motor preparation field, and motor field. The second dimension of the field is initially (i.e., before training) not associated with a specific feature, but instead provides redundancy in the existing representation to allow further specialization through learning. The association field receives one of its inputs from the spatial input field. This input is organized topologically along the spatial dimension, and is homogeneous along the second dimension. That means that a localized peak of activation in the spatial input field induces a vertical ridge of activation at the corresponding spatial location in the association field. The lateral interactions (local excitation and global inhibition) will produce a localized peak of activation from this ridge-like input (see Figure 2A).

**Figure 3:**
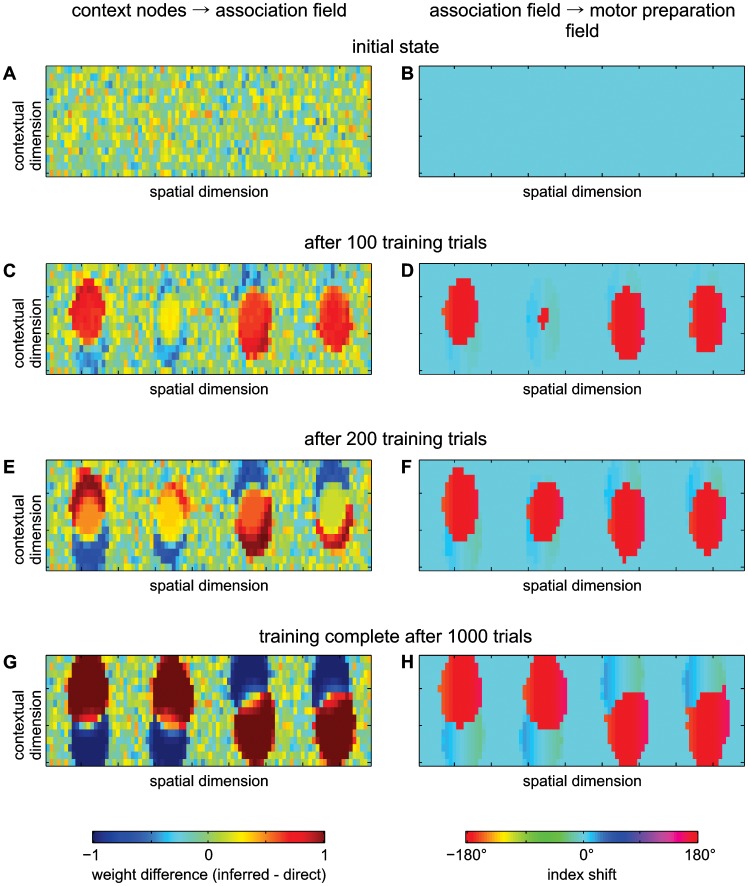
Weight changes in the model during IR training. (A, C, E, G) Weight difference matrix from the context input nodes to the association field. The color at each point of the field indicates the difference of the weights from the inferred context input node and the direct context input node to that point in the association field. In the untrained network, weight differences are randomly distributed around 0 without any spatial pattern (A). Over the course of IR training, distinct areas sensitive for direct or inferred context input evolve at the trained spatial positions (C, E, G). (B, D, F, H) Index shift in the projection from the association field to the motor preparation field (difference between spatial position of a point in the association field and the position in the motor preparation field to which it projects most strongly). In the beginning each point in the same spatial column preferably connects to the corresponding spatial position in the motor preparation field (B, index shift = 0°). After IR training those areas which prefer the inferred context input preferably connect to the opposite spatial position in the motor preparation field, corresponding to an index shift of about 180° (D, F, H).

The association field receives a second input from a set of two *context input nodes*. These nodes provide a simple, discrete representation of the context for the current trial (direct or inferred), indicated by the color cue. The nodes feature self-excitation and mutual inhibition, such that if one node becomes sufficiently activated by external input, it will remain active and suppress activation of the other node. There is an all-to-all connection from the context input nodes to the association field, which is initially unspecific (with small, random weights from each node to every point in the field, shown in Figure 3A). These connections are modified during the learning phase as detailed below, and can then influence where along the spatial input ridge an activation peak will form.

When a peak has formed in the association field, it remains stable even without exogenous inputs due to strong self-excitation. This peak provides a second input to the motor preparation field. The projection from the association to the motor preparation field is initially topologically organized along the spatial dimension, so that it supports a delayed reach movement to the memorized location of a previously presented spatial cue, but it is likewise subject to learning.

### Learning in the DNF model

The projections from the context neurons to the association field and from the association field to the motor preparation field are adapted according to a reward-driven Hebbian learning rule [24], [39]–[41]. We use two variants of the basic Hebbian rule that incorporate an implicit limit of weight growth, the ‘instar’ and ‘outstar’ learning rules in the formulation of Marshall [42]. These rules have successfully been used in topographical dynamic neural networks that are comparable to DNFs [43], [44]. We further adapted them to be used in a reward-dependent manner: As in the original rules, the weights between active regions are strengthened if the reward signal is positive, but in addition they are weakened if the reward signal is negative. Physiologically, a teaching signal could be conveyed by dopaminergic neurons via the cortico-basal circuitry (for review see [45]). It has been shown that dopamine neurons signal rewards through phasic activity and lack of expected reward through depressed activity [46]. However, our teaching signal does not habituate. Instead, we manually stop the learning process once the task has been trained.

The learning rules are applied once for each trial after the system has selected a response, which is defined as a sufficiently strong peak in the motor field (smoothed field output at one position exceeds a threshold 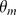). The direction of the planned reach, given by the position of the activation peak in the motor field, is compared to the rewarded goal location according to the task requirements. The trial is considered a success, with a reward signal of 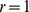, if the reach direction falls within a tolerance window (±8°) around the desired goal direction, and a failure, with a reward signal of 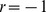, otherwise (this corresponds to the omission of the actual reward in the electrophysiological study).

The connection weights from the context neurons to the association field are updated according to the reward-dependent instar rule:
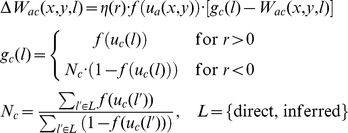



Here, 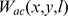 is the weight from the context node 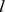 to position 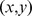 in the association field, 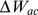 is the change of that weight in one trial, 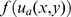 is the association field output at position 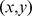 and 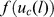 the output of context node 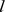. The learning rate 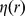 depends on the reward signal 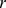 for that trial, and takes a larger value 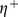 for 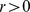 and a smaller value 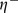 for 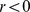. In the case of negative reward signal, a normalization is introduced to ensure that the overall weight changes are comparable for successful and fail trials.

With the instar learning rule, only those neurons in the association field that are active during a trial adapt their incoming connection weights from the context nodes. In the case of a positive reward signal, the weights of these neurons are adapted in such a way that the weight patterns become more similar to the current output pattern of the set of context nodes. The neurons whose weights have been adapted will be driven more strongly if the same output pattern of the context nodes appears again in subsequent trials, and will receive proportionally less input from different output patterns of the context nodes. Note that there is no normalization on the presynaptic side, such that multiple regions in the association field can form preference for the same context input without competition between them. This means that the instar rule supports development of divergent projections from the context nodes to the association field.

The weights from the association field to the motor preparation field are adapted according to the reward-dependent outstar rule:
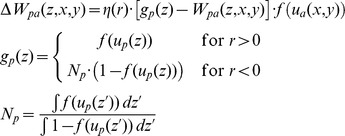



Analogously, 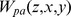 is the weight from position 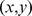 in the association field to position 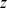 in the motor preparation field, 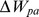 is the change of that weight, and 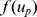 is the output of the motor preparation field.

With the outstar rule, the normalization of the weights is reversed compared to the instar rule. Again, weights are only adapted for those neurons in the association field that are active in a given trial (these are now the presynaptic neurons). If the reward signal is positive, outgoing weights of these neurons are adapted in such a way that the weight patterns become more similar to the postsynaptic output pattern in the motor preparation field, which reflects the actually performed reach. In the case of failed trial with negative reward signal, the connections from the active regions in the association field to the active region in the motor preparation field is weakened and the projection to all inactive regions is strengthened. This increases the probability that a different motor response is chosen in the next trial with the same conditions. Due to the normalization in this learning rule, each region in the association field can only strongly support a single motor response, but different regions may support the same response without competition between them. This means that the outstar rule supports development of convergent projections from the association field to the motor preparation field.

### Variations of the spatial goal selection task

In the first step, we will use our model to reproduce and explain the behavioral and neural observations of a previous monkey experiment, in which the authors investigated neural selectivity in the frontoparietal cortex during selection of rule-based spatial motor-goals [5]. The following scenarios were implemented to simulate this experiment.

#### Inferred-reach (IR) training task

Due to the pre-structuring, our model by default (without further training) produces ‘direct’ reaches, i.e., reaches to the spatial cue location. We trained the model to perform both direct and inferred reaches depending on the context (Figure 1C). At the beginning of an IR training trial the contextual cue and the spatial cue at one of four possible locations were presented. A second spatial cue (target cue) indicating the correct movement goal location for this trial was shown briefly at the end of the memory period. The movement goal could be either at the original spatial cue location (direct trial) or diametrically opposite to it, i.e. at a relative direction of +180° in the circular spatial input dimension (inferred trial). A ‘go’-signal was given directly after the presentation of the target cue to initiate a movement to it. In the monkey study the ‘go’-signal was indicated by turning off the manual fixation stimulus at the screen center, in the model this is simplified by disinhibition of the motor field. By this procedure in combination with the learning algorithm, the spatial cue was associated with either of two separate movement goals depending on the context (see Results). Direct and inferred trials were randomly intermixed with a predominance of inferred trials (80%) if not indicated otherwise. This large proportion of inferred trials emulates the over-exposure to inferred reach trials compared to direct reach trials during the behavioral training in the monkey study. We additionally analyzed the effects of different trial statistics on the model behavior in a systematic fashion (see Results). Over the course of training with a total of 1000 trials, the salience of the target cue (amplitude of input to the spatial input field) was reduced linearly from 1 to 0 so that eventually the network performed the context-specific mapping without presence of a target cue. This closely emulates the training procedure of the monkeys, although there the number of required trials was monkey specific and typically higher. Before any of the other task variants was applied to the model, it was first trained with this task, to be able to perform context-dependent mapping. We will refer to this as ‘IR-trained model’. This inferred reach training procedure was equivalent to the training procedure used in monkeys that learned the task [5]. (Note, though, that monkey training typically requires many smaller short-term adjustments of trial parameters to account for motivational factors and to optimize the training progress of the animal.)

#### Definite Motor-Goal task (DMG)

This task was used as a control condition to test if the model properly had learned to make direct and inferred choices depending on the context during IR training. In the DMG task the spatial and the contextual cue were presented simultaneously at the beginning of the memory period (Figure 1A). The cues were only presented briefly and the relative presentation times, compared to the memory period, were chosen to be equivalent to the physiological study (see Text S1 and [5]). The two mapping rules and four locations were presented with equal probability. The learning rate was set to zero in this control condition, since it was intended to probe the network state rather than to change it with this task.

#### Potential motor-goal task with context instruction (PMG-CI)

This task was used to examine the ongoing decision making process in situations with incomplete information (partial pre-cueing). It was a variation of the DMG task in which the spatial and the contextual cues were separated in time (Figure 1B). First only the spatial cue was presented. Therefore two potentially rewarded motor goals remained equally possible throughout the memory period, either at the location of the spatial cue or diametrically opposite to it. The contextual cue that was presented at the end of the memory period resolved this ambiguity and specified which of the two locations would be the rewarded motor goal. During testing in this task the learning rate was set to zero.

#### Potential motor-goal task with no context instruction (PMG-NC)

We used the PMG-NC task to test the free-choice behavior of the model. PMG-NC trials were identical to PMG-CI trials, except that no context instruction was shown at all. In this case two different reward schedules decided about which trials were rewarded and which not (see below). When the model was trained with PMG-NC trials, these were randomly interspersed with PMG-CI trials (PMG-NC:PMG-CI ratio 40∶60), equivalent to the monkey experiment.

#### Reward schedules

In the PMG-NC trials, two algorithms determined which of the two potential motor goals would be rewarded. In the *equal probability reward schedule* (EPRS) both potential locations were rewarded with equal probability (50∶50), irrespective of the choices of the model. From a game theoretical perspective the situation is equivalent to a matching pennies game in which the computer's strategy corresponds to the Nash equilibrium. With this reward schedule, we did not expect changes to any a-priori choice preferences that might have been present. This is because the expected reward is independent of the choices and neither behavioral strategy leads to more than 50% reward. In the *bias minimizing reward schedule* (BMRS) the success history was taken into account to decide which motor goal would be rewarded. Any behavioral bias for one of the motor goals was punished by lowering the probability of reward for that goal, so that the behavioral strategy that yields the highest reward ( = 50%) is one in which both motor goals are chosen with equal probability (for details see [5]).

### Monkey behavioral and electrophysiological data

The monkey behavioral and neuronal data which we refer to in this study are taken from a previous electrophysiological study and are described in detail elsewhere [5]. Previously unpublished behavioral data is presented from one of the same monkeys to test predictions of the model (see section Generalization in Results).

## Results

### Learning an arbitrary mapping rule

During the IR training task the model acquired the initially unknown inferred mapping rule, in addition to the default direct mapping. The model forms the required stimulus-response associations in the following way: The spatial cue induces an activation peak at the corresponding location in the association field, and at the same time in the motor preparation field (via the direct pathway). The association field peak remains self-sustained after the input disappears, and keeps supporting the activation in the motor preparation field, due to the a-priori topology of this projection (Figure 3B). The simultaneously presented contextual cue activates the corresponding context neuron, which likewise retains its activation through the neuron's self-excitation. In the trials early during IR training, a salient target cue then appears at the desired reach goal location. This new stimulus also drives activation in the motor preparation field via the direct pathway – for inferred trials at a location shifted by 180° from the original spatial cue location – and overrides the default reach plan which was induced by the first cue. In contrast, the memory peak in the association field remains largely unchanged, as it is stabilized by the lateral interactions, and suppresses the formation of new peaks. The movement onset is triggered at the end of the target cue presentation by a general disinhibition of the motor field (reflecting the disappearance of the central fixation stimulus in the monkey study). This forces the selection (activation) of a single location due to the strong inhibitory interactions in the motor field. In correct trials, i.e. when the surviving peak in the motor field ( = the selected reach) matches the goal location, the projections between active context nodes and active regions in the association field, as well as between active regions in the association and the motor preparation field, are strengthened, while others are weakened. Over the course of learning, the initially random connections from the context neurons to the association field (Figure 3A) are replaced by a more specific connection pattern: Early during training, patches with a preference for the inferred context form for the trained cue directions in the association field, dominating the central part of the context dimension due to the high proportion of inferred training trials (Figure 3C). For these patches, the connection weights to the motor preparation field are changed accordingly, such that the original topological projections to the motor preparation field (Figure 3B) have shifted by 180° to implement the inferred reach response (Figure 3D). Subsequently, patches with a preference for the direct context also appear, which retain the original topological projections to the motor preparation field. Through repeated reinforcement of initially random variations in the association peak positions, and under the influence of the lateral interactions in the fields, these coherent patches self-organize along the context dimension for each of the trained cue directions (Figures 3E, G). The projections to the motor preparation field keep adapting to reflect the context preferences of different regions in the association field (Figures 3F, H). The spatial positions that had not been trained (i.e. spatial locations at which cues had never appeared) do neither show a shift of their projections to the motor preparation field, nor do they show sensitivity for one of the context inputs.

The IR trained model was then tested in the DMG task. It reached a performance of 99% (n = 4000). This successful training confirms that the model can perform both the direct and inferred reach in a flexible context-depending manner, by re-learning local associations. To test whether this architecture and its integrated learning mechanism can solve a more general class of tasks, we also tested the system with a larger number of different contexts, all indicating different mapping rules. For three different contexts (with associated rotations of 0°, 180°, and 90°), the model still reached a performance of 94% in the DMG task after an analogous training procedure. For four contexts (indicating required rotations of 0°, 180°, 90° and 270°), a performance of 90% was reached (n = 4000). The decrease in performance for a higher number of different contexts is a consequence of interference between different context preferences in the association field: If the number of contexts becomes too high, the context specific regions that form during learning are no longer cleanly separated, and the corresponding projections to the motor preparation field do not form correctly. This limitation could be overcome in the model either by making the interactions in the associations field sharper (decreasing the kernel width in the context dimension) or by increasing the field size along the context dimension. In a biological system, the former would correspond to a sharpening of tuning properties of the neurons and the latter would correspond to the recruitment of a larger number of neurons for the association task.

### Selectively impaired generalization in monkey and model

**Figure 4:**
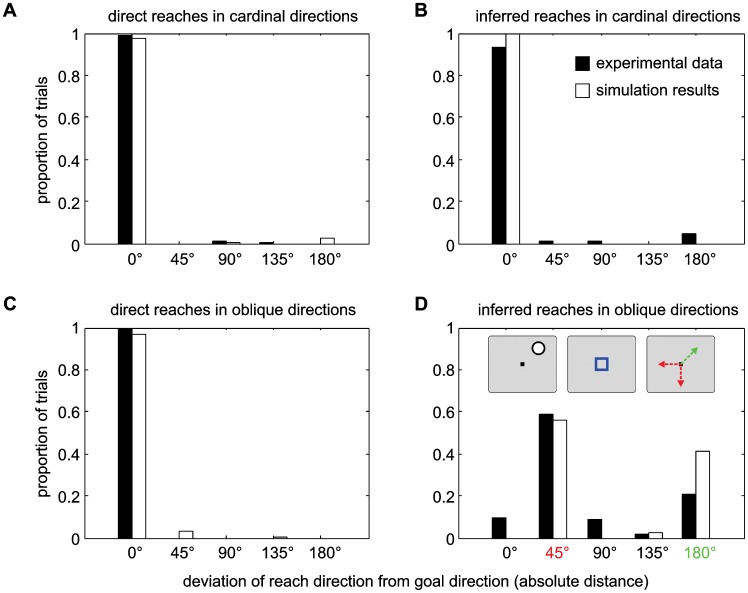
Generalization performance in monkey and model. Reaches performed by monkey (black) and model (white) were analyzed when generalizing from cardinal to oblique spatial cue directions. Bars show proportion of reaches in a direction relative to the rewarded goal (this means, 0° reaches are directed towards the correct goal, all others are failed reaches). Direct reaches to cardinal (A) and oblique (C) goals are almost always performed correctly. Inferred reaches to trained (cardinal) goals (B) are also almost always performed correctly, as was to be expected. If inferred reaches were required to oblique positions (D), both monkey and model show a similar pattern of failed reaches, illustrated in the inset of panel (D): Most reaches were made either in a previously trained cardinal direction adjacent to the goal direction (red, deviation of 45°) or in the direction of the spatial cue, meaning that a direct reach was performed (green, deviation of 180° from the goal direction).

A mechanism which learns spatial transformations via local associations instead of global geometrical rules is limited in its ability to generalize to new cue locations. We tested the generalization limits of our model and compared it to that of a monkey that performed the same task. The model was IR-trained with four spatial cue locations (e.g. the four cardinal directions) as described before. The model was then tested with four novel cue locations at positions between the trained locations (oblique directions). The model was unable to fully generalize and perform the task to the new locations. Importantly, the model made specific goal selection errors (Figure 4). Trials with a direct context cue to trained cardinal directions were not impaired (Figure 4a), and generalized to oblique goals with little performance deficits (Figure 4c). This is not surprising, given the pre-existing default mapping via the direct pathway. Inferred generalization trials, instead, showed a particular error pattern: In inferred trials to the trained cardinal directions the model also showed errorless performance (Figure 4b). Yet, in trials to an oblique inferred goal, the model either performed reaches to the direct reach goal (context error, approx. 40% of trials), or to a learned inferred reach goal at an adjacent cardinal direction (adjacent direction error, approx. 60%; Figure 4d).

The monkey control experiment was performed accordingly (previously unpublished data from one monkey). After learning context-specific direct and inferred reaches (as described in Methods) to four cardinal directions over the extended period of several weeks, the monkey was then tested with four oblique cue positions. Our reasoning was that the monkey should be able to generalize to the new locations with relative ease if the behavior was learned as a general, abstract rule. Conversely, if the inferred mapping was learned through local associations, proper performance should be restricted to the trained locations. The result was that the monkey performance remained high in blocks of trials in which the cardinal directions were used in either context (>90%, Figure 4A, B), or the oblique directions were used in the direct context (>95%, Figure 4C). But performance was clearly reduced in blocks in which the oblique directions were used in an inferred context (Figure 4D). The same two dominant types of errors as in the model could be observed in the monkey: >20% context errors and approx. 60% adjacent direction errors.

**Figure 5:**
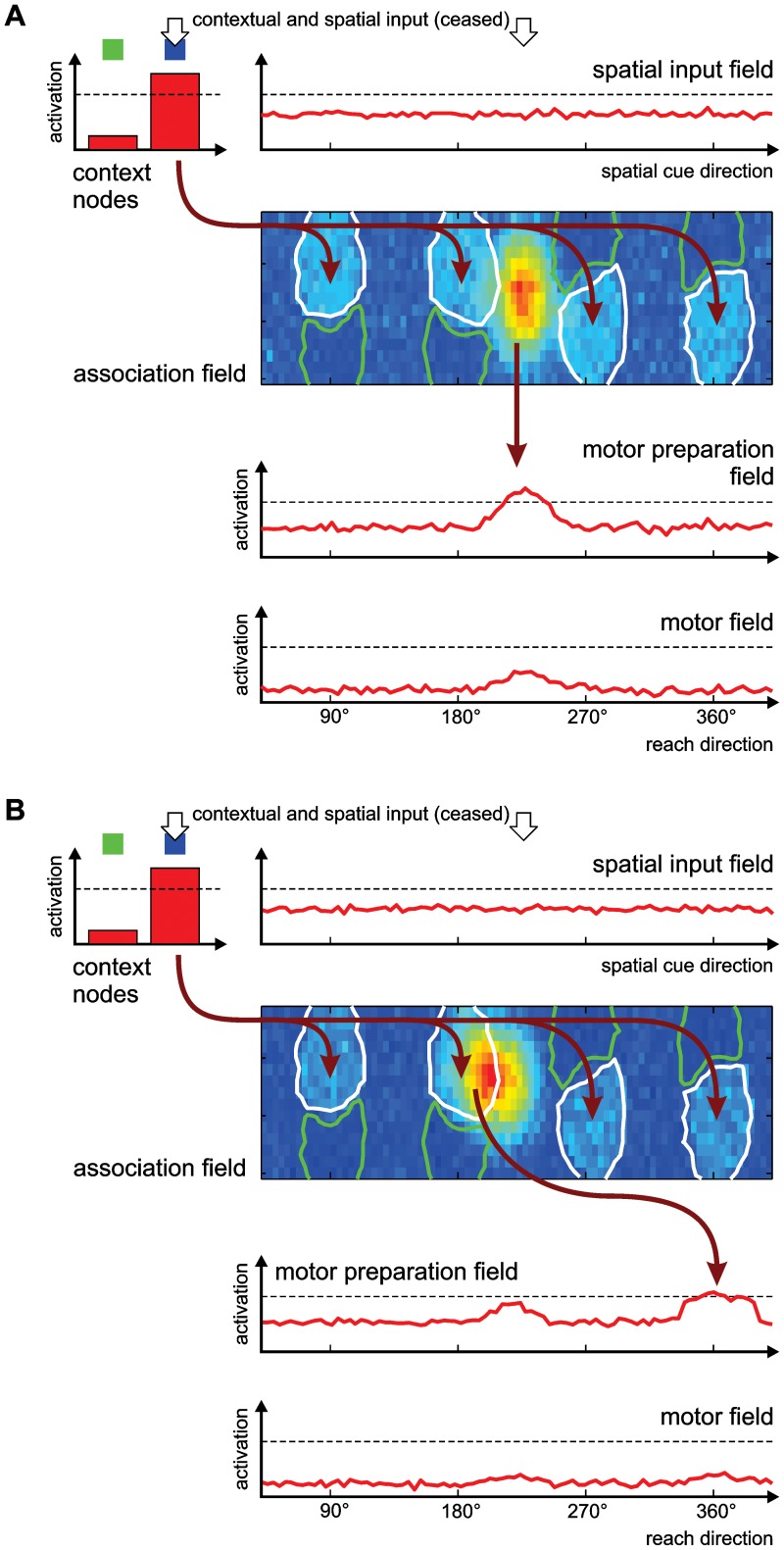
Origin of generalization errors in the model. Two snapshots of the activation patterns in the model during the memory period are shown, taken from different trials that developed different movement plans due to random noise in the model. In both cases, the spatial cue was located at 225° (an oblique direction not used during training), the blue context input indicates that an inferred reach should be performed. The model is depicted in the same form as in Figure 1. Arrows show the dominant active projections between fields that arise from the current activation patterns. Regions with pronounced preference for one context are outlined in the association field (green for direct context, white for inferred). (A) When the spatial cue was presented at the beginning of the trial (white arrow), it created an activation peak in the association field at the untrained oblique direction. This active region in the association field projects topologically to the motor preparation field, therefore preparing a reach to the spatial cue direction. This corresponds to a deviation of 180° from the goal direction, since the context cue indicates that an inferred reach should be performed. (B) If the activation peak in the association overlaps partly with a region that is selective for the inferred context, the activation peak may shift over to that region (the figure shows an intermediate step of this shift). This is driven by the input from the context node. The region of the association field that is now active has adapted its projection to the motor preparation field during training, and induces a new activation peak in the motor preparation field around 360°. This yields a deviation of 45° from the goal location, since the model now prepares one of the trained reaches in a cardinal direction.

In summary, in the way we implemented a context-specific mapping task via local association learning in our model, it predicted specific spatial generalization errors which we could confirm in the monkey behavior. The model provides a mechanistic explanation for these particular error types. As detailed in the previous section, the association field has formed two context-specific regions for each trained spatial cue direction. This is the result of the Hebbian learning. The regions which are specific for the inferred context project to the reach field at a position opposite to the spatial cue direction. If spatial input arrives from the spatial input layer for an untrained direction, together with an inferred-context signal from the context input nodes, it will create a peak between two of the context specific sub-regions in the association field (Figure 5A). This peak, which is self-sustained without exogenous input, may remain stable at this location. In this case, its projection to the motor preparation field will be centered on the position at which the spatial cue was presented (since the direct projection is the default before learning). This will result in a direct reach instead of the instructed inferred reach (context error). Alternatively, the peak in the association field may shift during the memory period to one of the regions that are selective for the ‘inferred’ context. These regions are moderately activated by the input from the context node, and if the activation peak slightly overlaps with one of them, it can get pulled towards it (Figure 5B). These regions implement the ‘inferred’ projection to the opposite direction in the motor preparation field, but only for the trained cardinal directions. The result will therefore be a reach in an inferred direction that is adjacent to the goal direction (adjacent direction error). The ratio of these two types of errors is determined by the ratio between the size of the field and the width of the lateral interactions.

We note that the adjacent direction error can also occur in oblique trials with the ‘direct’ context signal, and does appear in the simulation results in a small proportion of trials (Figure 4C, approx. 3% of trials). As in the inferred trials, this error is caused by a shifting of the peak in the association field from the untrained oblique direction to a trained cardinal direction under the influence of the input from the context nodes. However, the regions in the association field with a ‘direct’ preference are smaller than those with an ‘inferred’ preference, and typically situated at the borders of the field. They are therefore much less likely to overlap with the peaks that form in the oblique trials. Nonetheless, this type of error cannot be completely precluded.

### Adapting choice preferences to reward schedules

A core idea of our approach is that the mechanisms which are implemented for learning sensorimotor associations allow the network to also adapt its reward-based choice behavior. We tested this by confronting an IR-trained model with different reward schedules. To emulate the scenario of the previous monkey experiment [5], we picked a specific constellation of reward schedules, but the results are not restricted to this case.

**Figure 6:**
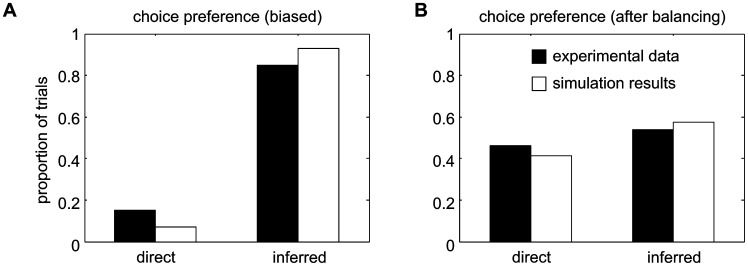
Choice behavior of monkeys and model in PMG-NC trials. If no context instruction is given in a trial, both model and monkeys show an inherent bias to perform the inferred reach after training (A). A balanced choice behavior (B) can be achieved by application of an appropriate reward schedule (BRMS).

After IR-training, the model is capable not only of correctly performing DMG trials, but also instructed trials in which the context cue appears later than the spatial cue (PMG-CI trials). In these trials, the model achieved 92% (n = 4000) correct choices (monkey performance in electrophysiological study was >98%). We then probed the model's free-choice behavior by presenting a spatial cue but no context cue (PMG-NC trials). Results show that our training procedure induces an inherent bias (93%) for inferred choices in free-choice situations (see below for systematic analysis of this effect), like was the case in the monkeys (85%±2% inferred trials; Figure 6A). For probing the inherent bias we used the equal-probability reward schedule (EPRS, see Methods), which creates no incentive to change the choice behavior.

**Figure 7:**
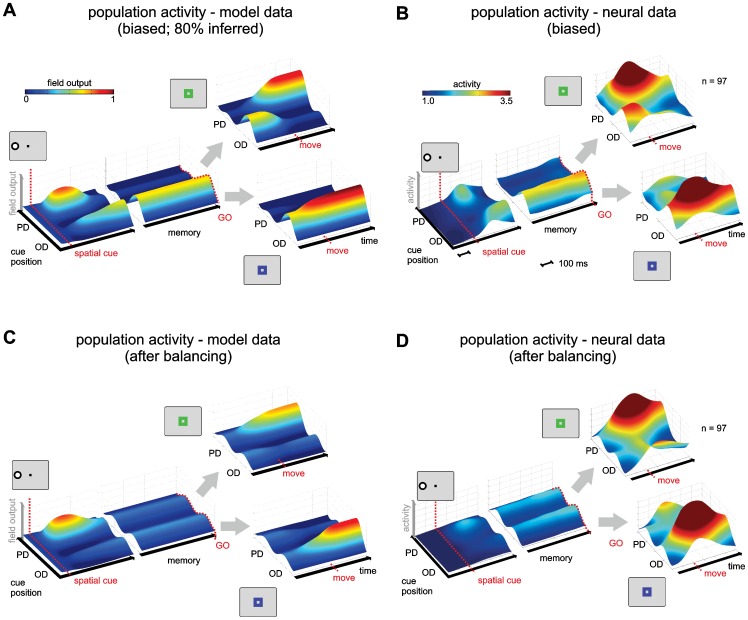
Comparison of population activation in model and electrophysiological data. Plots show the averaged and normalized field output from the motor preparation field in the model (A, C) and from electrophysiological recordings in PRR (B, D) during the PMG task. Prior to averaging and normalizing, the real and model neurons' selectivity profiles were aligned according to their preferred directions in DMG trials (PD: preferred direction, OD: opposite-to-preferred direction). The averaged and normalized activity of real neurons during the PMG task in the biased (B) and balanced (D) datasets is shown for three epochs, aligned to cue onset, ‘go’-signal, and movement onset, since the length of the epochs was variable. The model neurons were aligned accordingly even though the epochs had fixed lengths. It can be seen that during the memory period in the model and in the real data plots, only one activation ridge is stable throughout the memory period, before a bias minimizing reward schedule (BMRS; see Methods) was introduced (A, B). After application of the BMRS, two stable ridges with a lower activation remain during the memory period (C, D).

The bias for selecting inferred reaches is also apparent in the output pattern of the motor preparation field in the model (Figure 7A), which qualitatively reproduces the observed neural activity in monkeys' PRR (Figure 7B): When the spatial cue is presented, activation initially rises for the direction of this cue (corresponding to the preparation of a direct reach). In the model, this is the result of the direct pathway from the spatial input field to the motor preparation field. However, this direct plan is quickly replaced by activation for the inferred reach (in the opposite direction), and this activation remains throughout the memory period. If a context cue is given at the end of the memory period (PMG-CI trials), the activation in the motor preparation field can undergo another change: If the cue for the direct context is given, the field activation rises strongly for the direction of the spatial cue (the rewarded goal direction for this case), and the activation supporting the inferred reach ceases. If the cue for the inferred context is presented, the peak of activation retains its position, and rises further when the motor response is selected. We will further investigate the underlying mechanism for the bias in the following section.

We then switched to a bias-minimizing reward schedule (BMRS). The model parameters (connection weights) that had developed in the previous testing phase were taken as starting conditions. The model developed a balanced choice behavior under the new reward schedule (Figure 6B; model: 41% direct reaches, n = 4000; monkeys: 46%±3% direct reaches), and, correspondingly, the two potential motor goals are equally represented in the motor preparation field during the memory period (Figure 7C). These two effects were also seen in the monkey data (Figure 7D). The adaptation to the new reward schedule confirms – as we expected based on the reward-dependent learning rule that is used – that the DNF model is capable of adapting its choice behavior to increase its overall reward probability, in a fashion that is consistent with the experimental data.

### Input statistics during association learning bias free-choice behavior

A major implication of an overlapping neural substrate and shared learning mechanism for sensorimotor association learning and reward-based action selection is that the learning history must inevitably influence the choice behavior. A surprising finding in our previous monkey study was the strong bias of the well-trained monkeys to almost exclusively prepare and execute the inferred reach in free-choice situations with EPRS reward. We hypothesized that this bias arose from the higher number of inferred reach trials during early training [5]. Similar effects can also be observed in human behavior [47]. We used our adaptive DNF model to show how the reward-dependent Hebbian-type learning can reproduce this bias in the decision process as an effect of the input statistics during training.

**Figure 8:**
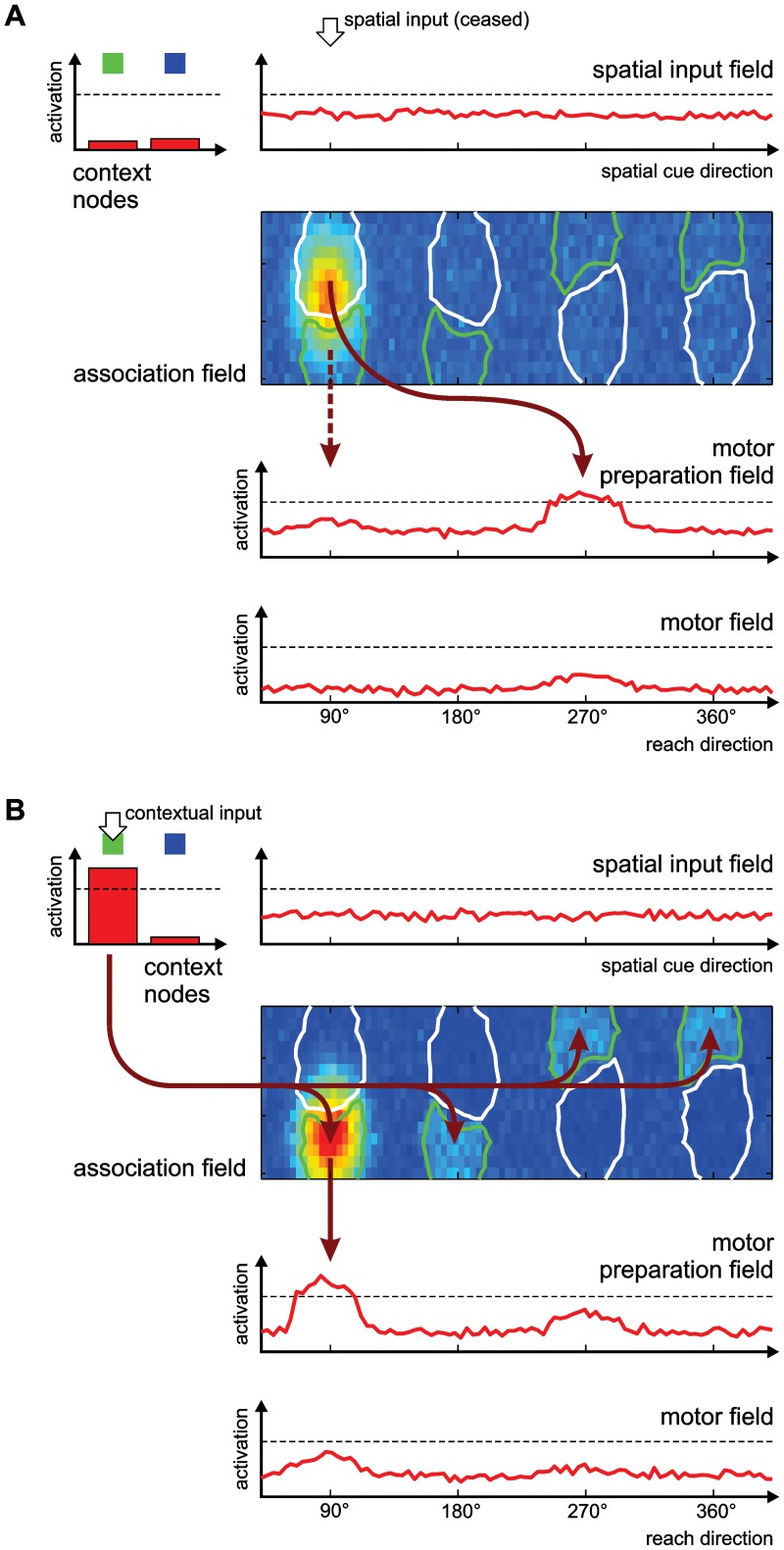
Emergence of bias for inferred reaches in the DNF model. The figure shows two snapshots of the activation patterns in the model during a single PMG trial. (A) During the memory period, after the presentation of a spatial cue, an activation peak has formed in the association field. Its position along the spatial axis reflects the direction of the spatial cue, while its location along the second dimension is unspecific and spans both context-sensitive regions (shown as outlines in the association field, green for direct, white for inferred context). The region that shows preference for the inferred context is substantially larger than the direct-context region, due to the high proportion of inferred trials during training. This region projects to the location in the motor preparation field which codes for a reach in the direction opposite to the spatial cue. The competitive interactions in the motor preparation field further amplify this stronger input that supports the inferred reach. (B) When a context signal for a direct trial is given at the end of the memory period, the context input induces a shift of the peak in the association field: It is pulled almost completely onto the region specific for the direct context with which it partly overlapped. The input to the motor preparation field changes accordingly, leading to a switch in that field's activation pattern and a stronger activation of the ‘direct’ reach direction.

In the model, the initial presentation of the spatial cue induces the formation of a sustained activation peak in the association field (Figure 8A). In the absence of context input, e.g. in the memory period of PMG trials, the separate regions with different context preferences do not influence the activation distribution in the DNF, and the peak typically spans both regions. In trials with later context instruction (PMG-CI), the subsequent presentation of a context input changes the attractor states of the DNF, and the activation peak shifts towards the region that has a preference for the given context (Figure 8B). The projections from that region to the motor preparation field then select the appropriate action. In free-choice trials without context instruction (PMG-NC), the choice behavior depends on the connectivity structure which was imposed by the earlier training.

As presented above, when the model is trained with a ratio of 80% inferred trials to emulate the intense inferred reach training procedure in the electrophysiological study, it develops a bias to prepare the inferred reach in PMG trials, with a time course of activation in the motor preparation field that qualitatively reproduces the recorded neural activity in monkeys (Figure 7A, B). The cause for this bias in the model is that the regions in the association field that have developed a preference for the inferred context are substantially larger than those for the direct context, as a result of the inferred reach being performed more frequently during training (see Figure 3b). When the activation peak in this field forms before the presentation of the context cue, it typically covers a larger area of the inferred-context region (Figure 8A), resulting in a stronger projection to the opposite reach direction in the motor preparation field. The competitive interactions in the motor preparation field then suppress the activation for the weakly excited direct reach direction.

**Figure 9:**
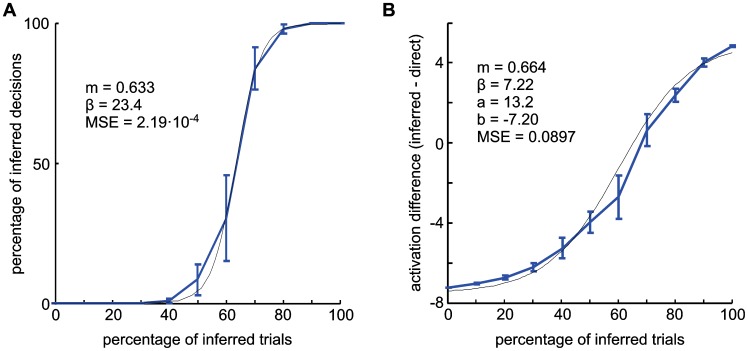
Influence of input statistics on model behavior and activation pattern during the memory period. (A) The behavioral bias for inferred reaches in the free-choice trials depends on the percentage of inferred trials during IR training and rises continuously in a sigmoidal fashion (logistic fit function; black curve). (B) The difference of the mean activation of the motor preparation field at the preferred and opposite-to-preferred position during the memory period shows a softer, but also approximately sigmoid increase when the number of inferred trials is increased.

We systematically varied the ratio of direct to inferred trials during the IR training in the model and tested the resulting spatial response profiles in the motor preparation field and the choice behavior in PMG-NC trials (Figure 9). The number of inferred choices increases continuously with the ratio of inferred trials during IR training (Figure 9A), in an approximately sigmoid fashion (logistic function fit: m = 0.633, β = 23.4; MSE = 2.19 * 10^−4^). Note that the sigmoid curve is not centered at 50% inferred training trials, but at close to 60%. This is an effect of the direct reaches being the default action before training, implemented by the initial connection pattern from the association field to the motor preparation field. The difference in the underlying activation strength for inferred versus direct goal representations in the motor preparation field during the memory period also increases monotonically and in an approximately sigmoid fashion with the fraction of inferred trials during IR learning (Figure 9B, fit with a scaled and shifted sigmoid curve, 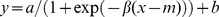: m = 0.664, β = 7.22, a = 13.2, b = −7.20, MSE = 0.0897).

Note that this result is indeed an effect of the input statistics, not of the expected reward for different choices. Even in training sets with 100% reward rate for both direct and inferred reaches the described biases still developed in the model (data not shown).

## Discussion

Neurophysiological data suggests that learning of sensorimotor associations, decision making, and movement planning share a common neural substrate, that includes frontoparietal sensorimotor areas [2], [4]–[6], [15], [21], [48]–[53]. If this is the case, the competitive interactions that underlie the selection between alternative action plans (and thereby decision making) are no longer independent from the process of learning sensorimotor associations. With our DNF model we demonstrate how learning of new spatial visuomotor mappings and adaptation to changing reward schedules in decision making can be achieved via a reward-driven Hebbian learning rule within the same substrate. In a free-choice task, our learning model adapted its choice behavior to two distinct reward schedules. In a generalization task, it predicted specific patterns of instructed choice errors, as we could confirm in a behavioral monkey control experiment. When the reward was independent of the choice, the input statistics during the initial learning of the different sensorimotor mappings determined the behavioral choice preferences in later free-choice situations. In summary, the long-term sensorimotor learning history and the short-term reward history are both critical variables which determine behavioral choice preferences in motor-goal selection tasks and which are linked by the same underlying neural mechanisms.

### Relationship to neurophysiology

Delineating a 1-to-1 correspondence between our model and the neurophysiological functional architecture of the primate brain can obviously only be coarse for several reasons. For example, it is yet unclear to a large extent, how many, which and in which way brain areas (cortical and subcortical) contribute to such high-level tasks as context-dependent, rule-based, and reward-driven visuomotor reach-goal selection. For example, similar task-related neural activation patterns during spatial goal selection tasks can be found in parietal and in premotor areas (e.g. [5], [10]), with the mutual roles of these areas not being clear yet. Also, similarity in neural activation patterns during manual and ocular selection tasks suggests that equivalent mechanisms are implemented in the oculomotor systems [21], [54], [55], yet both systems comprise different cortical and subcortical structures. The intra- or interareal connectivity pattern of the recorded neurons is typically not available with the currently available recording techniques, and many other areas in the cerebral cortex are not explored yet at the required level of detail provided by single cell electrophysiology. These factors impair model validation.

Yet, the model should be seen as a rough sketch of cortical frontoparietal visuomotor processing. The spatial input field is retinocentrically organized, and mimics the organization of extrastriatal visual cortex and the available dorsal-stream visual input to the frontoparietal reach network via areas V6/V6a in the parieto-occipital sulcus [56], [57]. The context input field provides a simplified color/rule representation. This input reflects the currently valid mapping rule and could originate in frontal lobe areas (e.g., dorsolateral prefrontal cortex, PFC) in which the task-relevant stimulus features have already been extracted, and the task rule rather than the actual color of the stimulus is represented [58], [59]. On the output side of the model, the motor preparation and the motor field employ a population code over possible movement directions. Such encoding of a movement plan or motor preparatory signals is based on neural population activity patterns during reach preparation in cortical motor areas [60], [61]. The direct pathway in our model can be seen as reflection of the forward projection along the dorsal visual stream and via dorsal premotor cortex to the primary motor cortex. Alternatively, the direct pathway could be motivated by low-level integration paths [62], e.g., the retinotectal visual pathway in case of saccadic tasks, which can bypass cerebrocortical processing especially during stimulus-triggered oculomotor behavior (for review see [63]). To draw connections to empirical findings in our specific task, we compare the field output of the motor preparation field to electrophysiological data from the posterior parietal cortex [5], but we note that the premotor cortex shows very similar activity patterns [5], [10]. We assume that the motor preparation field provides a functional representation that might be anatomically distributed over the frontoparietal sensorimotor cortex. The motor field could be equated to parts of the primary motor cortex (M1) and caudal parts of PMd. The implemented gating mechanism in the motor field (gain change as result of ‘go’ cue processing), might be a function provided by subcortical structures. It has been suggested that modulation of motor activity similar to a gating mechanism, i.e. facilitation or inhibition, could be provided by the basal ganglia via the so called ‘direct’ or ‘indirect’ pathway (not to be confused with our use of the terms); for review see [64].

The indirect pathway in our model allows for flexible, context-specific, goal-directed behavior. The two-dimensional association field, which implements the working memory and the actual rule learning, is reminiscent of processing in the cortico-basal loops between PFC and the premotor cortex (PMC) with the basal ganglia [65]–[68]. Certain aspects of the association field could also be localized in the frontoparietal loop, since especially PMd was shown to be relevant for learning abstract visuomotor associations (for example see [6], [48]–[52]). Also, the development of a combined selectivity for reach direction and context input is consistent with a gain modulation by context described for neurons in PMC and posterior parietal cortex (PPC) areas [26]. We note that the second (context) dimension of the association field in the model is initially simply providing redundancy in the representation, and only through the learning process it takes on the role of separating different context preferences. Corresponding redundancies may well exist in pre-motor areas in the cortex, such that the combined direction/context selectivity can develop through specialization of neural response properties. We assume that similar redundancies would also exist in the other representations in the model, but are not made explicit since they are not critical for the model's behavior.

### Learning arbitrary remapping rules through local associations

When we designed the adaptive DNF architecture, we assumed that the behavior of the monkeys in the experiment did not rely on an explicit representation of a geometrical transformation rule to achieve the visuomotor mapping, but rather on specific associations between individual stimulus combinations and the rewarded motor response. This may at first seem counterintuitive for a task that can be described unambiguously through a simple rule. It should be noted, however, that from a computational perspective the forming of concrete associations (which can be achieved by established mechanisms like Hebbian learning) is much more straightforward than the recognition and implementation of a general rule.

Our assumption was supported by the control experiment, in which the monkey had to generalize the learned mapping “rule” to untrained positions. If the monkey applied a geometric transformation rule, one would have expected easy generalizing to novel goal directions. Instead, the monkey showed a highly specific pattern of errors that the model was able to predict, and which in the model was an emergent effect of the local association learning. Note that the observed failed reaches could not be explained by a failure of the monkeys to identify the proper context, since direct trials in all directions and inferred trials in cardinal directions were conducted correctly. Instead, the associated motor responses to the untrained oblique goal positions in the inferred context were undefined. This led to responses which were either guided by the default behavior (a seeming ‘context’ error), or which resulted in the selection of a neighboring trained motor association (adjacent direction error). These observations suggest that the context-dependent reach task in monkeys was not learned through the application of a general mapping rule to the spatial cue positions, but rather by individual, local associations between the spatial and context cue and the rewarded reach location.

The adaptable DNF model implements such association learning in that it develops specialized attractor states in the association field, with dedicated sub-regions which prefer different mapping “rules”. In this implementation the context errors originate in the initial topological connection pattern from the association field to the motor preparation field, which is still prevailing after the IR-training for those spatial cue directions that have not been trained. The adjacent direction errors can be explained by a spread of activation from such untrained regions in the association field to neighboring sub-regions which were affected by the IR training and are now associated with the inferred context.

In the model, the adjacent direction error also occurs in small percentage of the oblique direct trials, due to the same mechanism. The fact that this error is not observed in the experimental data may indicate that some aspect of the task is not fully captured by the computational model. For instance, the representation and processing of the direct and inferred context signals may not be as symmetrical in the biological system as it is in the model. In particular, the context signal might affect the processing more globally, e.g. by strengthening the direct pathway for the ‘direct’ context cue and the indirect pathway for the ‘inferred’ context cue. This would decrease the impact of training certain directions on the behavior in direct trials. Such a mechanism of executive control has previously been employed in a DNF model of task switching [69].

We note that this implementation of the spatial mapping rule in the association field does in principle not have to be locally restricted. If the sub-regions that implement the ‘inferred’ mapping were expanded over the whole spatial dimension, and if their projections to the motor preparation field were changing in a more continuous fashion, they could implement the general mapping rule for arbitrary spatial cue directions. Forming such a connection pattern would require a sufficiently large number of training directions, which would provide the necessary fine sampling of the directional space to generalize the mapping rule to all directions through averaging. Conversely, the model in its current state is not capable of generalization in a stricter sense, such as the transfer of a rule to completely novel stimuli. Introducing such capabilities would require a substantial extension of the current architecture.

This does not mean that the mechanism we presented cannot also be involved in the learning of abstract rules. It is conceivable (e.g. in the case of humans performing this task) that generalized connection patterns as described above for different mapping rules accumulate and prevail in the system. Learning a specific variation of a mapping task then only requires the association of the context cue with the appropriate known mapping. This would allow a fast generalization from few examples. In general, however, we propose that the learning via local associations may be the default case, and that forming of true generalizations is an extension that builds on previously learned associations and additional neural structures.

### Integration of learning and action selection

With the adaptive DNF model, we integrate two behavioral functions in a single neural architecture. On the one hand, we provide a process model of movement plan formation and action selection. It is in this respect similar to another recent modeling study of decision making in the fronto-parietal cortex [2]. It extends this previous approach to allow the selection of motor goals that were not explicitly spatially cued (inferred reaches) in a context-dependent manner. On the other hand, the model also incorporates a learning mechanism that allows it to acquire new visuomotor associations and thereby at the same time become adaptable to different reward schedules in ambiguous choice situations. The learning mechanism allows a close emulation of the training procedure in the monkeys. In particular, it does not require an explicit teaching signal for the desired motor response, as has been used in neural network models of the same task [22]. Instead, the desired behavioral response is shaped by using a second visual cue (which is processed by the system in the same way as other visual cues in the task) and reinforced through reward. Other theoretical accounts that focus on the learning process deal only with a small number (typically just two) of possible response choices, represented by discrete nodes [24], [40], [70], [71]. They are therefore less suited to capture the process of action selection from a continuous space of motor acts in the fronto-parietal network, and could not possibly explain the resulting consequences for the generalization behavior that we found. Most of these models also do not investigate behavioral biases and free choice tasks, although Soltani and Wang [72] showed how the posterior probability for a choice alternative being rewarded, given a set of cues, could be computed by synapses trained with a reward-dependent learning rule comparable to the one used in our system. Again, this model dealt only with a two-alternative choice and the cues independently predicted the rewarded choice, whereas in our task two different types of cues must be combined to determine the rewarded response.

### Influence of reward contingencies and input statistics

A reward-driven Hebbian learning algorithm enables the model to adapt to changes in the reward schedule in a manner similar to what is called the ‘matching law’. This means, biases in the reward schedule can produce biases in choice behavior and thereby adapt the choice to the reward probabilities [11], [73], [74]. Since the model learns the sensorimotor associations and the reward contingencies via the same projections, the model's sensitivity to the reward history in free-choice trials interacts with its learned associations, and vice versa. For example, if the ratio of free-choice trials is very high, it can happen that the model ‘unlearns’ the initially trained mapping because the context-sensitivity of the association field and the conjunction of the context with specific projections to the motor preparation field slowly decay in the free-choice trials (data not shown). The observation that errors can cancel the learned mapping has similarly been made in a model by Fusi et al. [24]. Reducing the learning rates after initial learning of multiple associations would slow such unlearning process but also decrease the sensitivity to changing reward schedules. If the susceptibility to changing reward schedules in free-choice should stay high, then it is necessary to also present regular instructed trials along with the free-choice trials to preserve the learned associations, which is what we did here and in our previous monkey experiments.

Interestingly, in free-choice tasks which do not encourage balanced behavior (i.e., choice-independent reward schedules like our EPRS), the learning algorithm can easily lead to a biased behavior. Even small imbalances in the probabilities of either choice can self-enhance the probability of the same choice in later trials. This is especially true if the reward probability is high (e.g. 100%), in which case an initially randomly chosen option will be more likely to be chosen again. Such a behavioral bias in free-choice trials is evident in our electrophysiological study [5] and has also been reported in other studies [16], [75].

Not only does the free-choice reward learning affect the learned associations, but, vice versa, the input statistics during learning of the stimulus-response associations also have an impact on the free-choice behavior, as our model results show. For example, the model's free-choice behavior can be biased even if the model is perfectly able to solve the instructed tasks. Humans rely on prior probabilities if they have to base their decision on lacking or ambiguous evidence [47], [76]–[78]. From a Bayesian point of view, the activation distribution in our model during the memory period of PMG trials can be interpreted as a representation of the prior distribution. If in a potential motor goal trial no further information is provided, the model decides according to this prior distribution. If further evidence is provided, as is the case at the end of the memory period in our instructed trials, then the prior distribution is over-ruled (Figure 7). Since the probabilities for direct and inferred trials were equal during the recording experiments in the electrophysiological study, but the monkeys still both showed the same strong bias in favor of inferred reaches, we assume that the inferred bias was acquired during the training of the task, when more inferred than direct trials were presented (unpublished observation).

### Conclusions and predictions

Our model successfully integrates sensorimotor processing and working memory formation with decision making. The reward-driven Hebbian learning mechanism which we use for learning context dependent visuomotor mappings is sufficient to also explain adaptation to probabilistic reward contingencies and at the same time creates susceptibility for input statistics during learning. With our model we could reproduce the electrophysiological results from a previous study [5], which showed a similar dependency on reward contingencies. Since continuous reward-driven neuronal weight adaptations change the behavior in free-choice trials, we can also explain how manipulations of the reward schedule produce any ratio of biased behavior, as has been observed in other physiological studies [11], [73], [74] and could be the source of matching behavior in foraging tasks. From this integrated approach, we can also provide a concept for how biased behaviors in decision tasks can emerge from the learning history of the system.

## Supporting Information

Table S1 **Field parameters.** Tabular summary of parameters that were used for the experiments.
(PDF)

Table S2 **Connection strengths between the fields.** Tabular summary of the parameters for the connection strength between the neural fields used in the model.
(PDF)

Text S1 **Formal description of the neurodynamic model.** The file gives the full dynamic equations of all neural fields in the model, the equations for the learning mechanism, and the methods used for the evaluation of the model behavior.
(PDF)
