# Supplementary material for: Sensorimotor Learning Biases Choice Behavior: A Learning Neural Field Model for Decision Making
Source: PLoS Comput Biol. 2012 Nov 15;8(11):e1002774. doi: 10.1371/journal.pcbi.1002774 (PMC3499253; doi:10.1371/journal.pcbi.1002774)
Supplement: Table S1 — Field parameters. Tabular summary of parameters that were used for the experiments. (PDF) [file pcbi.1002774.s001.pdf]

Table S1: Field parameters

field	resting level h	noise level q	lateral excitation c^{exc}	lateral/global inhibition c^{inh} or c^{gi}
s	-3	0.1	7.5	5
c	-3	0.05	10	2.5
a	-3	0.2	22.5	0.125
p	-2	0.1	4	0.25
m	-6	0.1	20	1
