# Supplementary material for: Sensorimotor Learning Biases Choice Behavior: A Learning Neural Field Model for Decision Making
Source: PLoS Comput Biol. 2012 Nov 15;8(11):e1002774. doi: 10.1371/journal.pcbi.1002774 (PMC3499253; doi:10.1371/journal.pcbi.1002774)
Supplement: Table S2 — Connection strengths between the fields. Tabular summary of the parameters for the connection strength between the neural fields used in the model. (PDF) [file pcbi.1002774.s002.pdf]

Table S2: Connection strengths

connection	strength *w*
as	5
ac	4
ps	5
pa	0.075
mp	3
pm, exc	9
pm, gi	0.1
