# Supplementary material for: Sensorimotor Learning Biases Choice Behavior: A Learning Neural Field Model for Decision Making
Source: PLoS Comput Biol. 2012 Nov 15;8(11):e1002774. doi: 10.1371/journal.pcbi.1002774 (PMC3499253; doi:10.1371/journal.pcbi.1002774)
Supplement: Text S1 — Formal description of the neurodynamic model. The file gives the full dynamic equations of all neural fields in the model, the equations for the learning mechanism, and the methods used for the evaluation of the model behavior. (PDF) [file pcbi.1002774.s003.pdf]

Text S 1: Formal description of the neurodynamic model

For the formal description of the DNF model, we identify each field or set of nodes and its associated parameters with a single letter index: s for the spatial input field, c for the context input nodes, a for the association field, p for the motor preparation field and m for the motor field. The spatial dimension of the fields always spans the circular space of possible directions for spatial cues and motor responses (0° to 360°). For the numerical simulations, this continuous space is sampled by 88 discrete units. The context dimension of the association field is sampled by 15 discrete units.

All fields use the same output function *f*, that is applied to the field activation $u(\vec{x})$, with a steepness parameter $\beta = 1$:

$$f\big(u(\vec{x})\big) = \frac{1}{1 + \exp\big(-\beta u(\vec{x})\big)}$$

Interactions (both within and between fields) are mediated by an interaction kernel k that is convolved with the field output, using the general convolution operation in one or two dimensions,

$$[k * f(u)](\vec{x}) = \int_{\mathbb{R}^n} k(\vec{x} - \vec{x}')f\big(u(\vec{x}')\big)d\vec{x}'.$$

We use a narrow Gaussian kernel k_{exc} for all excitatory interactions and a broader Gaussian kernel k_{inh} for lateral inhibitory interactions, with the general form

$$k(\vec{x}) = \frac{1}{(2\pi\sigma^2)^{\frac{n}{2}}} \exp\left(\frac{|\vec{x}|^2}{2\sigma^2}\right)$$

for $n = 1$ or 2 dimensions. The kernel widths used for all fields (given in the discrete field units) are $\sigma_{exc} = 2.5$ and $\sigma_{inh} = 7.5$. The field parameters and connection strengths are given in Tables S1 and S2.

The activation u_s of the one-dimensional spatial input field is governed by the differential equation

$$\tau\dot{u}_s(x) = -u_s(x) + h_s + w_{ss}^{exc}[k_{exc} * f(u_s)](x) - w_{ss}^{inh}[k_{inh} * f(u_s)](x) + I_s(x)$$

with time constant $\tau = 40ms$ (that is shared by all fields) and resting level h_s. The lateral interactions consist of local excitation with strength w_{ss}^{exc} and surround inhibition with strength w_{ss}^{inh}, and the field receives external input I_s that reflects the positions of the spatial cues. Each spatial cue is modeled through a Gaussian input of strength $w_s = 6$ and width $\sigma_s = \sigma_{exc} = 2.5$, centered on the direction in which the spatial cue is presented. In the inferred reach training, the strength the second (target) cue starts at $w_s = 6$ and is gradually reduced to 0 over the course of the training. Note that we omitted the dependence of activation on time in this and the following equations for the sake of conciseness.

The activation u_c of the two context nodes for the possible contexts $z \in Z = \{\text{direct}, \text{inferred}\}$ is described by the differential equation

$$\tau\dot{u}_c(z) = -u_c(z) + w_{cc}^{exc} f\big(u_c(z)\big) - w_{cc}^{gi} \sum_{z' \in Z} f\big(u_c(z')\big) + I_c(z).$$

Each node excites itself with a strength w_{cc}^{exc} and both nodes contribute to a global inhibition signal with strength w_{cc}^{gi}. When a context signal is given, the external input I_c to the corresponding node is set to $w_c = 6$.

The association field spans two dimensions, the directional spatial dimension as in the spatial input field and a second dimension that provides redundancy to learn the context-depending mapping. The field equation for this field is

$$\tau \dot{u}_a(x,y) = -u_a(x,y) + h_a + v_a(x) + w_{aa}^{exc}[k_{exc} * f(u_a)](x,y) - w_{aa}^{gi} \iint f\big(u_a(x',y')\big) dx'dy' + w_{as}[k_{exc} * f(u_s)](x) + w_{ac} \sum_{z \in Z} W_{ac}(x,y,z) f\big(u_c(z)\big).$$

Lateral interactions consist of local excitation and global inhibition. The projections it receives from the spatial input field are localized along the spatial dimension and homogeneous along the second dimension. The inputs from the context nodes to each point in the field are described by a learned weight matrix W_{ac} (with entries limited to the range $[0,1]$) and a global strength w_{ac}. In addition, there is a constant preshape (or memory trace) in the field reflecting all spatial cue directions A used in one experiment. It is defined as

$$v_a(x) = \sum_{\alpha \in A} c_v \exp\left(-\frac{(x-\alpha)^2}{2\sigma_v^2}\right),$$

with $c_v = 0.5$ and $\sigma_v = 3$.

The activation u_p of the motor preparation field is described by the equation

$$\tau \dot{u}_p(x) = -u_p(x) + h_p + w_{pp}^{exc}\big[k_{exc} * f(u_p)\big](x) - w_{pp}^{gi} \int f\left(u_p(x')\right) dx' + w_{ps}[k_{exc} * f(u_s)](x) + w_{pa} \iint W_{pa}(x,x',y') f\big(u_a(x',y')\big)\, dx'dy' + w_{pm}^{exc}[k_{exc} * f(u_m)](x) - w_{pm}^{gi} \int f\big(u_m(x')\big) dx'.$$

The field features local excitation and global inhibition, and receives inputs from three sources: The projection along the direct pathway from the spatial input field, the projection from the association field, and a feedback projection from the motor field. The projection from the association field uses a weight matrix W_{pa} that is subject to learning and a global weight w_{pa}. The feedback from the motor field contains a local excitatory and a global inhibitory component, to ensure that the selected reach direction is also reflected in the activation pattern of the motor preparation field.

The field equation for the motor field is given by

$$\tau \dot{u}_m(x) = -u_m(x) + w_{mm}^{exc}[k_{exc} * f(u_m)](x) - w_{mm}^{gi} \int f\big(u_m(x')\big) dx' + w_{mp}\big[k_{exc} * f(u_p)\big](x) + b_m.$$

The strong lateral interactions (local excitation and global inhibition) ensure that only a single activation peak can form at any time from the (potentially multimodal) motor preparation field input, yielding a uniquely specified direction for a reach movement. When the 'go'-signal is given, the global boost value b_m is set to 6, prior to that it is zero. A movement is considered to be initiated when the convolved motor field output $[k_{exc} * f(u_m)]$ exceeds a threshold $\theta_m = 0.75$ at any position. The selected reach direction ϕ is then determined as the (circular) center of mass of the field output at this time:

$$\phi = \mathrm{atan2}\left(\int f\big(u_m(x)\big) \sin(x)\, dx, \int f\big(u_m(x)\big) \cos(x)\, dx\right)$$

Numerical simulations of the fields were performed using the Euler method, with discrete time steps of $2ms$. A stochastic element is introduced by adding a normally distributed random noise term $q\xi(t)$ to the activation of at each point of a field and to each node, where $\xi(t)$ is drawn from a random distribution $\mathcal{N}(0,1)$ in every time step. The noise strength q is set to 0.1 by default, but is decreased for the context node (since they lack the spatial interactions that have an averaging effect on the noise) and increased for the association field (since we assume that this more plastic, higher-level structure is more strongly exposed to unspecific inputs). The architecture was instantiated 10 times, using a random number

generator with different seeds to produce the random initialization of the weight matrix W_{ac} and the random noise in each field.

DMG trials consisted of a brief pre-cue phase, then the presentation of the spatial and context cue for 200ms (during which the stimulus inputs to the spatial input field and context input nodes were activated), followed by an 800ms memory period and then the boost of the motor field to initiate the selection of a motor response. In PMG trials with context instruction, the context cue was presented during the last 200ms of the memory period, as was the spatial target cue in the IR training trials.

The update of adaptable connection weights takes place once for every training trial when a reach movement is initiated. The model response is then evaluated (correct if the distance between reach direction and goal direction is $\leq 8°$, failed otherwise) and the reward for the trial assigned ($r = 1$ for correct, $r = -1$ for failed). For completeness, we provide the learning rules here again (see also main text). The weight $W_{ac}(x, y, l)$ from context node l to a position (x, y) in the association field is updated according to the reward-dependent instar rule:

$$\Delta W_{ac}(x,y,l) = \eta(r) \cdot f\big(u_c(x,y)\big) \cdot [g_c(l) - W_{ac}(x,y,l)]$$

$$g_c(l) = \begin{cases} f\big(u_c(l)\big) & \text{for } r > 0 \\ N_c \cdot (1 - f\big(u_c(l)\big) & \text{for } r < 0 \end{cases}$$

$$N_c = \frac{\sum_{l' \in L} f\big(u_c(l')\big)}{\sum_{l' \in L}\Big(1 - f\big(u_c(l')\big)\Big)}$$

The reward-dependent learning rate is

$$\eta(r) = \begin{cases} 0.1 & \text{for } r > 0 \\ 0.05 & \text{for } r < 0 \end{cases}$$

The weight $W_{pa}(z, x, y)$ from position (x, y) in the association field to position z in the motor preparation field is updated according to the reward-dependent outstar rule:

$$\Delta W_{pa}(z,x,y) = \eta(r) \cdot \big[g_p(z) - W_{pa}(z,x,y)\big] \cdot f\big(u_a(x,y)\big)$$

$$g_p(z) = \begin{cases} f\big(u_p(z)\big) & \text{for } r > 0 \\ N_p \cdot (1 - f\Big(u_p(l)\Big) & \text{for } r < 0 \end{cases}$$

$$N_p = \frac{\int f\Big(u_p(z')\Big)\,dz'}{\int 1 - f\Big(u_p(z')\Big)\,dz'}$$
